# Notes on Two Hundred and Twelve Consecutive Operations in Four Thousand Six Hundred and Ten Cases among the Medical Patients, Bristol Royal Infirmary

**Published:** 1901-09

**Authors:** E. H. Edwards Stack

**Affiliations:** House Physician.


### NOTES ON TWO HUNDRED AND TWELVE CONSECUTIVE OPERATIONS IN FOUR THOUSAND SIX HUNDRED AND TEN CASES AMONG THE MEDICAL PATIENTS, BRISTOL ROYAL INFIRMARY.

BY

E. H. EDWARDS STACK, M.B. Cantab, F.R.C.S.,

*House Physician.*

---

IN any hospital there are always on the medical side a number of minor operations, if, indeed, they should be dignified by the name of operations at all. There is, for instance, the use of the exploring needle in a chest, pericardium or pleura, in the abdomen, in a joint, or a swelling, and in the spinal canal; further, in some of these cases an aspirator or a trocar is used to take off a considerable amount of fluid as a treatment of the disease. In other cases, an abscess forms in an otherwise medical case, as quinsy, and is not of sufficient surgical importance to necessitate the calling in of the surgeon; or, again, operation for urgent dyspnœa, which often arises in medical cases, is in many hospitals, on account of custom or convenience, consigned to the house physician, as are also incisions in the legs, or the use of Southey's trocars for œdema. Bleeding, from time immemorial, has been regarded as belonging to the domain of medicine.

It is about such cases as these that I have arranged some tables, and wish to make a few remarks. They include as many as I have been able to collect by going over the last four years' medical notes at the Royal Infirmary; and I wish to thank the physicians in that institution for permission to use them. The reading through notes of 4,610 cases taken in many different handwritings, and some of them very indifferent, takes a very considerable amount of time and patience, and I have no doubt that I may have overlooked some of them, as, of course, there is no index to these minor points, and possibly some may have even been omitted from the notes. Still, I think there cannot be many that are not here recorded.

Diphtheria, necessitating tracheotomy or intubation, of which there have been over thirty cases, has been omitted, and for two reasons. In the first place they are more distinctly surgical, and in the second, I hope at a future date to make a more detailed account of them. Suffice it to say here, that I am quite convinced that intubation for diphtheria is, since the introduction of antitoxin, much the best procedure in the majority of cases treated in hospital.

Exploration of the chest is always performed to assist or confirm a diagnosis before aspiration or incision is resorted to. No one, I think, ever regrets that he has put a needle into the chest, even if the result has been negative; but who can say that he has not been sorry in some cases that he has not done it sooner? I believe that in the whole of medical literature there are but very few cases recorded where the slightest untoward result has followed its proper use, so few that one never hesitates on that account to have recourse to it should there be any indication; but many differ as to when there is an indication. I believe the indication might be stated thus: If in any case the knowledge gained by putting in a needle could have any effect in changing for the better the treatment of the patient, then it should be done, even though the chances of gaining that knowledge be very small. For instance, in a case practically known to be a pleural effusion, where there is the faintest possibility of that effusion being purulent, then the question ought at once to be settled, as there is no doubt that the most important factor for a rapid cure of an empyema is the earliness of the date at which the pus is evacuated. It has been said that there is a danger of converting, by the introduction of septic matter, a serous effusion into a purulent one. Were this so, then it would be an argument for withholding the exploring syringe. Now I wish to lay particular stress on the negation of this point. I would go so far as to say that it almost never occurs, for the following reasons:—

1. There is no doubt that effusions of serum often contain micro-organisms, though it is very rare to find tubercle bacilli in the fluid; still, they have, with care, often been found. I have myself several times found the diplococcus pneumoniæ in a clear serous effusion; and if these micro-organisms are present, and causing the irritation they are known to do, then they can at a later date cause sufficient irritation to induce an exudation of leucocytes sufficient to make the fluid purulent. This I proved in one case lately. The patient had pneumonia, and at the same time an effusion, which was quite clear, but of an acute nature, as evidenced by the thick clot of fibrin which took place in the syringe when it had been left for a short while. This fluid contained the pneumococcus, from which pure cultures on agar were obtained. A few days later another syringeful was extracted. This time it was slightly turbid, and again contained only the same organism. A third time it was quite purulent, and no further organism was discovered. The patient had a pneumonic crisis, but the temperature did not remain down, and a few days later, the day of the third exploration, he was operated on for empyema. Had this case not been watched carefully from a bacteriological point of view, one might easily have assumed that it had been infected by the first exploration.

2. The needle which is introduced, if septic, has the organisms either on its external surface or inside; and it seems to me to stand to reason that if they were on its exterior, it would be the tissues through which it passes that would be affected; and, if interiorly, they would be drawn back with the fluid into the barrel, and so not infect the effusion. I have never known any inflammation take place in the tissues, and have not heard of any case in which it had done so.

3. I have known many cases that have been tapped again and again with an aspirator, and they have, without exception, remained sterile to the end.

I have gone thus fully into this point, not because there is a case against it, but because it is important that this danger (so called) should be dispelled from the minds of those who may happen, from the fear of it, to withhold their hand when the patient would be benefited by an exploration, paracentesis, or even incision. Everyone will agree as to the necessity for using antiseptic precautions; they are no additional trouble, and, even if they are not necessary, may just as well be used.

Owing to the difficulty of following all cases to a termination, and as their number is only small, it is impossible to prove much by actual figures; but, taking my impressions for what they are worth, the following are the conclusions I have arrived at:—

1. That there is practically no contra-indication to exploring a chest on the slightest pretext, not the slightest ill-effect following it in over a hundred consecutive performances.

2. That occasionally an unexpected result on exploring will much help in the further treatment or prognosis of the case.

3. That the cases most benefited by aspiration are acute effusions, without signs of other disease; and the sooner this is done the sooner the cure.

4. That in less acute cases the cure is hastened, though paracentesis may have to be employed a second or third time.

5. That the finding of pus with subsequent evacuation gives a good result, in direct ratio to the early period at which it is discovered, especially after cases of pneumonia.

6. That aspiration in cases due to heart disease and Bright's disease, is of more benefit in the former than the latter, and often of but little use in either.

7. That the indications for stopping aspiration are lividity, cough, and distress.

8. That it is often better to cease aspiration for a few minutes now and then, to allow the displaced parts to

*TABLE OF TWENTY-NINE CASES OF PARACENTESIS OF THE CHEST IN BRISTOL ROYAL INFIRMARY.*

| Medical Volume. | Folio. | Sex. | Age. | Disease. | Ounces withdrawn. | Cured in wks. after Operation | Remarks. |
|---|---|---|---|---|---|---|---|
| 49 | 677 | M | 22 | Pleuritis | S. 30 | 4 | Still poor entry of air. |
| | 797 | F | 48 | Sarcoma of Ovaries, Hydrothorax & Ascites | S. 16 | ... | Much relieved. Death. |
| | 813 | F | 22 | Pleuritis | S. 78 | 8 | Remained dull for weeks. |
| 50 | 1050 | M | 14 | Pleuritis | S. 24 | 4 | Improved. |
| | 1108 | F | 18 | Hydro-Pneumothorax | S. 63 | | |
| | | | | 6 months later | P. 10 | ... | Death from Phthisis. |
| | 1121 | M | 32 | Pleuritis | S. 70 | 3 | Much relieved. |
| 51 | 297 | M | 41 | Pleuritis | S. 10 | 2 | No change in 3 weeks before Paracentesis. |
| | 320 | M | 53 | Pleuritis | S. 80 | 4 | Blood stained. |
| | | | | ,, 14 days later | S. 50 | | ,, ,, |
| | 330 | F | 39 | Pleuritis | S. 50 | 4 | |
| | 571 | M | 63 | Malignant Disease of Pleura | S. 50 | ... | Tapped 16 times, always clear. Death. |
| 52 | 472 | F | 21 | Pleuritis | S. 70 | | |
| | | | | ,, 7 days later | S. 44 | 6 | |
| | 554 | F | 26 | Pleuritis | S. 30 | 3 | |
| | 572 | F | 60 | Mitral disease | S. 44 | ... | Did not re-accumulate. |
| 53 | 792 | F | 5 | Pleuritis | S. 26 | 6 | |
| | 860 | M | 67 | Sarcoma of lung | S. 12 | ... | Blood stained. Also had Hæmoptysis. |
| | | | | ,, later | S. 30 | ... | Clear. |
| | | | | ,, ,, | S. 15 | ... | Clear. Death. |
| | 900 | F | 18 | Pleuritis and Ascites | S. 41 | ... | Much relieved. |
| 54 | 240 | F | 21 | Pleuritis | S. 5 | 4 | No change for 3 months before aspiration. |
| 55 | 387 | M | 40 | Pleuritis | S. 90 | 3 | Much relieved. |
| 56 | 291 | M | 33 | Pleuritis | S 12 | 2 | |
| 57 | 302 | M | 18 | Pleuritis | S. 56 | 8 | Probably disease of lung as well. |
| 58 | 380 | M | 18 | Pleuritis | Sero purulent | 6 | Empyema later. |
| | 676 | M | 61 | Heart Failure | S. 20 | ... | Temporary relief. |
| 59 | 854 | M | 64 | Pneumonia and Effusion | S. 2 | ... | Not relieved. Death. |
| | 889 | F | 22 | Pleuritis | S. 14 | 4 | Much relieved. |
| | 988 | M | 13 | Pleuritis | S. 18 | 4 | |
| | | | | ,, later | S. 52 | ... | Much relieved. |
| 60 | 120 | M | 4 | Pneumonia, Empyema | P. 2 | 4 | Not relieved. Operation. Cured. |
| 61 | 551 | F | 38 | Pleuritis | S. 38 | 3 | Much relieved. |
| | 524 | F | 23 | Lympho-Sarcoma Lung | S. 48 | ... | Chylous-like fluid. |
| | | | | ,, ,, later | S. 20 | ... | Relieved temporarily. |
| | | | | ,, ,, later | S. 22 | ... | Death. |
| 60 | 642 | M | 5 | Pleuritis | S. 8 | ... | Not relieved. Slow improvement. |

S Signifies Serum, and P Pus.

EXPLORATION OF THE CHEST IN FORTY-THREE CASES.

| Volume. | Folio. | Sex. | Age. | Disease. | Result of Exploration. | Remarks. |
|---|---|---|---|---|---|---|
| 49 | 691 | M | 4 | Empyema | P | Operation; cured in 3 months. |
| | 813 | F | 22 | Pleural effusion | Nil | Aspirated; 78 ounces. |
| 50 | 920 | M | 40 | Empyema | P | Operation; wall calcareous; death. |
| | 1108 | F | 18 | Hydro-Pneumothorax | Nil | Aspirated several times, serum; died 6 months later; empyema and phthisis. |
| | 1109 | M | 18 | Empyema | P | Operation; cured. |
| | 1121 | M | 42 | Pleuritis | S | Dull to apex; not aspirated; cured in 2 months. |
| 51 | 147 | M | 37 | Pneumonia | P | Operation; still discharging 9 months later. |
| | 306 | M | 36 | Pleuritis | Nil | No effect. |
| | 203 | F | 22 | Pleuritis | S | Heart to Rt. of Sternum on admission; not aspirated. Heart natural situation in 3 weeks, and patient cured. |
| 52 | 373 | M | 33 | Pneumonia | P | Operation; cured in 4 weeks. |
| | 379 | M | 44 | Pneumonia | Nil | Cleared up. |
| | 393 | F | 40 | Signs of Effusion | Nil | Cleared up. |
| | 408 | M | 2 | Empyema | P | Operation; ill 4 weeks on admission; died 3 days after. |
| | 482 | M | 5 | Empyema | P | Ill 6 weeks before admission; cured 6 weeks after. |
| | 976 | F | 3 mos. | Empyema | P | Brought in moribund; aspirated, 4 oz.; died same night. |
| 53 | 890 | M | 30 | Signs of Effusion | Nil | Cleared up. |
| 54 | 8 | M | 47 | Pleuritis | S | Been ill some months; cured in 3 months. |
| | 344 | M | 10 | Pleuritis | S | Cured in 5 weeks; not aspirated. |
| 55 | 415 | M | 15 | Pleuritis | S | Cured in 7 weeks; not aspirated. |
| | 444 | F | 13 | Phthisis | P | Died 5 days after exploration. |

| | | | | | | |
|---|---|---|---|---|---|---|
| | *508* | *F* | *3* | *Empyema ... ... ...* | *P* | *Operation; not relieved; death.* |
| | 945 | M | 11 | Pleuritis ... ... ... ... | Nil | Remained dull for months. |
| 57 | 137 | M | 9 | Empyema ... ... ... | P | Cured in 4 weeks. |
| | 293 | M | 9 | Pneumonia ... ... ... | Nil | Cleared up. |
| | 363 | M | 15 | Pneumonia ... ... ... | P | Operation. |
| 58 | 380 | M | 18 | Empyema ... ... ... | P | Operation; cured in 3 weeks. |
| | 385 | M | 3 | Empyema ... ... ... | P | Death in 10 days. |
| 59 | 755 | M | 13 | Empyema ... ... ... | P | Operation; cured. |
| | 788 | M | 3 | Pneumonia ... ... ... | P | Needle put in 7 places before pus found; operation. |
| | 796 | M | 32 | Pleuritis ... ... ... ... | S | Cleared up without aspiration. |
| | 834 | F | 4 | Empyema ... ... ... | P | Operation. |
| | 840 | F | 3 | Pneumonia ... ... ... | P | Operation. |
| | 921 | F | 21 | Pleuritis ... ... ... ... | S | Cleared up slowly without aspiration. |
| 60 | 138 | F | 10 | Pleuritis ... ... ... ... | S | Cleared up in 2 months. |
| | 248 | M | 56 | Pneumonia ... ... ... | P | Operation; cured in 3 months. |
| | 168 | M | 27 | Pneumonia ... ... ... | P | Operation. |
| | 207 | M | 5 | Pneumonia ... ... .. | Nil | Persisting dulness. |
| | 684 | M | 17 | Pneumonia ... ... ... | P | Operation. |
| | 704 | M | 17 | Pleuritis ... ... ... ... | S | Not aspirated; cured in 2 weeks. |
| 61 | 577 | F | 20 | Pleuritis ... ... ... ... | S | Not aspirated; cured in 5 weeks. |
| | 632 | M | 23 | Hydro-Pneumothorax ... | S | Signs of phthisis later. |
| | 663 | M | 40 | Pleuritis ... ... ... ... | Nil | |
| | 697 | M | 8 | Pneumonia ... ... ... | Nil | Probably basal phthisis. |

S Signifies Serum. P Pus.

Exploration was also performed in all the 29 cases of Aspiration of the Chest as a preliminary proceeding, making a total of 72 operations.

adapt themselves slowly to their new relations, and the lower the vacuum (consistent with a flow) the better.

9. That the patient can bear the operation much more easily if the feet are allowed to rest on a stool at the side of the bed than if he sits up with his feet still in the bed.

10. That persisting dulness after aspiration, the so-called "thickened pleura," is usually due to collapsed lung; and in these cases, getting the patient into the open air, and encouraging deep breathing, have a good effect.

11. That age or sex, or the amount of fluid present, has no marked effect on the ultimate prognosis.

12. That letting out the air in a pneumo-thorax when there is tension should be done, but its effect is usually only temporary.

13. That pleural effusion is not met with more at one time of the year than another.

14. That not very infrequently on exploring a chest nothing is found, when, as proved later, there is fluid present. The cause of this may be that the needle is too small or impervious, or it may not be far enough in; or, more often, it penetrates too far, engaging the lung, or it may miss the effusion by being beyond its limits, or a sufficient vacuum is not formed. Sometimes a little blood from pricking the lung will get into the needle, clot there, and prevent the fluid entering the needle, even when it is afterwards free in the fluid; or, in the same way, a bit of lymph may get in and block the needle. I have often, under these circumstances, pushed the piston home again, and so freed the needle, and though by doing this a little air may be injected, it does no harm.

15. That, if in one place fluid is not found, a good plan is to withdraw the needle till the point is again level with the parietal pleura, and then push it in again in a new direction—this causes very little pain—then, unscrew the barrel, push home the piston, and re-make the vacuum after again fixing on the barrel.

16. That there is, for practical purposes, no danger of bleeding from any wounded vessel.

17. That if there is a slight leak anywhere in the aspirator which cannot be remedied, a little ether in the aspirating bottle will prevent it filling with froth.

18. That brandy should be at hand, as the patient may occasionally become faint.

19. That when the flow ceases, moving the needle to a new spot (often lower down), or letting the patient lean towards the affected side, will often cause it to recommence.

20. That a displaced apex-beat seldom returns to quite its normal situation immediately. It often takes several weeks to do so, often never entirely returns, and sometimes goes beyond the normal in the opposite direction, and may remain there.

21. That pneumonia in adults is more likely to be followed by empyema in men than in women.

22. That although 70 per cent. of cases of pleuritis with effusion are said to be due to tubercle, in hospital practice, at any rate, it is quite rare to be able to prove the association of the two.

23. That empyema under the age of five years is a very fatal disease.

24. That pus in children in an empyema may be nearly clear at the apex, and quite thick at the base.

25. That empyema in adults is very rare, except after acute pneumonia.

26. That in the operation for empyema the removal of a piece of rib should be the rule at any age, and the reverse the exception.

27. That serum is not so uncommon in pneumo-thorax with liquid as is generally supposed, but it has a tendency to become purulent more than any other serous effusion.

*(To be continued.)*

